# Norepinephrine-Induced Calcium Signaling and Store-Operated Calcium Entry in Olfactory Bulb Astrocytes

**DOI:** 10.3389/fncel.2021.639754

**Published:** 2021-03-23

**Authors:** Timo Fischer, Jessica Prey, Lena Eschholz, Natalie Rotermund, Christian Lohr

**Affiliations:** Division of Neurophysiology, Department of Biology, Institute of Zoology, University of Hamburg, Hamburg, Germany

**Keywords:** olfactory bulb, astrocytes, norepinephrine receptors, calcium signaling, store-operated calcium entry

## Abstract

It is well-established that astrocytes respond to norepinephrine with cytosolic calcium rises in various brain areas, such as hippocampus or neocortex. However, less is known about the effect of norepinephrine on olfactory bulb astrocytes. In the present study, we used confocal calcium imaging and immunohistochemistry in mouse brain slices of the olfactory bulb, a brain region with a dense innervation of noradrenergic fibers, to investigate the calcium signaling evoked by norepinephrine in astrocytes. Our results show that application of norepinephrine leads to a cytosolic calcium rise in astrocytes which is independent of neuronal activity and mainly mediated by PLC/IP3-dependent internal calcium release. In addition, store-operated calcium entry (SOCE) contributes to the late phase of the response. Antagonists of both α1- and α2-adrenergic receptors, but not β-receptors, largely reduce the adrenergic calcium response, indicating that both α-receptor subtypes mediate norepinephrine-induced calcium transients in olfactory bulb astrocytes, whereas β-receptors do not contribute to the calcium transients.

## Introduction

Norepinephrine is one of the major neuromodulators in the mammalian central nervous system. It is involved in a variety of vital cognitive functions such as memory and attention (Berridge and Waterhouse, 2003; Aston-Jones and Cohen, 2005). Furthermore, it has been shown that norepinephrine plays a crucial role in sleep-wake cycle (O'Donnell et al., 2015). The locus coeruleus (LC) is the major site for the biosynthesis of norepinephrine. Its widespread neuronal projections innervate many different brain regions such as the neocortex, amygdala, hippocampus, and hypothalamus. They also reach the main olfactory bulb, where ≈40% of LC neurons project to (Shipley et al., 1985). Thus, noradrenergic input to the main olfactory bulb is part of the centrifugal innervation and plays a key role in odor learning, recognition, and recall (Linster et al., 2011). Previous studies showed that the majority of norepinephrine fibers target the external plexiform layer (EPL), the internal plexiform layer (IPL), and the granule cell layer (GCL), and to a lesser extent the mitral cell layer (ML) and the glomerular layer (GL) (McLean et al., 1989). Norepinephrine release in the main olfactory bulb regulates the strength of GABAergic inhibition of mitral cells depending on the norepinephrine receptor subtype activated (Trombley, 1992; Trombley and Shepherd, 1992; Nai et al., 2009). In accordance with the noradrenergic fiber distribution, each of the three major subtypes, α1-, α2-, and β-receptors, are expressed in multiple layers of the olfactory bulb, and distinct main olfactory bulb neurons seem to express various norepinephrinereceptor subtypes, e.g., granule cells express α1- and α2-receptors, whereas mitral cells express all three subtypes (Nai et al., 2009, 2010). While expression and function of norepinephrine receptors are well-studied in neurons, it is unknown to date whether olfactory bulb astrocytes possess noradrenergic receptors.

An increasing number of studies has unraveled the role of astrocytes in the brain during the past two decades and it is generally accepted that astrocytes act on neurons in a manner that extends the traditional view of being solely supporting cells. Astrocytes take part in the regulation of synaptic transmission and plasticity through a wide variety of processes, developing the idea of the tripartite synapse (Araque et al., 1999a,b; Brockhaus and Deitmer, 2002; Volterra and Meldolesi, 2005; Henneberger et al., 2010). Specifically, these processes include modulation of synaptic glutamate and potassium homeostasis, activity-dependent synaptogenesis and elimination, neurotransmitter synthesis, synaptic connectivity, and vascular function (Araque et al., 2014; Lohr et al., 2014; Guerra-Gomes et al., 2017). In this context, astrocytic calcium signaling provides a fundamental mechanism to integrate synaptic function of surrounding cellular circuits and has been subject to intense research in the last decades (Deitmer et al., 1998; Verkhratsky et al., 2012; Verkhratsky and Zorec, 2019). The effect of norepinephrine on astrocytes and astrocytic calcium signaling has been extensively studied in diverse brain regions such as sensory cortex, where it has been demonstrated that astrocytes play a key role in the modulatory network of norepinephrine (Vardjan and Zorec, 2017). However, although norepinephrine innervation in the olfactory bulb is high and has a strong influence on neuronal network activity, the role of norepinephrine on olfactory bulb astrocytes remains unknown. In this study, we investigated the physiology of norepinephrine receptors in olfactory bulb astrocytes by employing the calcium indicator Fluo-8 and confocal calcium imaging in acute olfactory bulb brain slices. Our results show that olfactory bulb astrocytes respond to bath application of norepinephrine with α-receptor-mediated calcium release from internal stores. Furthermore, we demonstrate the contribution of store-operated calcium entry (SOCE) to norepinephrine-induced calcium transients.

## Materials and Methods

### Animals Used for Slice Preparation

Naval Medical Research Institute (NMRI) outbred mice from postnatal day 7 (p7) to p21 were used for calcium imaging experiments. No differences in results were found within this time range. Mice were bred in the institute's animal facility at the University of Hamburg and all experiments were carried out in accordance with the recommendations of the European Union's and local animal welfare guidelines (03/2020; approved by Behörde für Gesundheit und Verbraucherschutz, Hamburg, Germany). Mice were anesthetized using isoflurane (5% mixed with 1 L/min O_2_) and decapitated before using for experiments. Olfactory bulb slices were prepared as described before (Fischer et al., 2012). Brains were quickly dissected and transferred into a chilled artificial cerebrospinal fluid (ACSF, see below). Two hundred micrometer thick horizontal slices of the bulbs were cut using a vibratome (Leica VT1200S, Bensheim, Germany). Slices were stored in ACSF for 30 min at 30°C and then at room temperature until starting experiments. Artificial cerebrospinal fluid was continuously gassed with carbogen (95% O_2_/5% CO_2_; buffered to pH 7.4 with CO_2_/bicarbonate).

### Solutions and Chemicals

The standard ACSF for acute brain slices contained (in mM): NaCl 125, KCl 2.5, CaCl_2_ 2, MgCl_2_ 1, D-glucose 25, NaHCO_3_ 26, NaHPO_4_ 1.25, gassed during the entire experiment with carbogen to adjust the pH to 7.4. Phosphate buffered solution (PBS) contained (in mM): NaCl 130, Na_2_HPO_4_ 7, NaH_2_PO_4_ 3. Norepinephrine and 2-APB (2-aminoethyl diphenylborinate) were obtained from Sigma-Aldrich (Darmstadt, Germany). ICI 118,551 hydrochloride (ICI), MPEP, CGP55845, MRS 2179, and ZM241385 were obtained from Tocris (Bristol, UK). BTP2 (YM-58483), D-APV, NBQX, gabazine, TTX (tetrodotoxin), CPA (cyclopiazonic acid), prazosin, and rauwolscine were obtained from Abcam (Cambridge, United Kingdom). All substances were stored as stock solutions according to the manufacturers' description.

### Immunohistochemistry

Immunohistochemistry on olfactory bulbs of NMRI mice (p14) was performed as described before (Klein et al., 2020). After preparation, olfactory bulb hemispheres were kept in PFA 4% for 1 h at room temperature. Hemispheres were cut into 100-μm sagittal slices using a vibratome (VT1000S, Leica, Nussloch, Germany) and then incubated for 1 h at room temperature in blocking solution (10% normal goat serum, 0.5% Triton X-100 in PBS). Subsequently, the slices were incubated with primary antibody solution, containing antibodies diluted in 1% NGS, 0.05% Triton X-100 in PBS for 48 h at 4°C. The primary antibodies chicken anti-NET (1:500, Synaptic Systems, Göttingen, Germany) and rabbit anti-GFAP (1:1,000, Dako, Hamburg, Germany) were used. Afterwards, slices were incubated in PBS with the following secondary antibodies for 24 h at 4°C: goat anti-chicken Alexa 488 (1:1,000, Abcam) and goat anti-rabbit Alexa 555 (1:1,000, Invitrogen Thermo Fisher, Darmstadt, Germany). Additionally, DAPI (5 μM, Thermo Fischer) was added to visualize nuclei. Slices were mounted on slides using a self-hardening embedding medium (Immu-Mount, Thermo Fisher). A confocal microscope (C1 Eclipse, Nikon, Düsseldorf, Germany) was used to image immunohistochemical staining. Image processing and analysis is described in Klein et al. (2020). Image stacks were obtained with an axial step size of 150 nm. Stacks were deconvolved using Huygen's software (SVI, Hilversum, Netherlands). Projections were made using Image J (NIH, Bethesda, USA), contrast and brightness were adjusted with Adobe Photoshop CS2 graphics editor.

### Calcium-Imaging

Slices were incubated with the membrane-permeable form of the calcium indicator Fluo-8 (Fluo-8-AM; 2 μM in ACSF) made from a 2 mM stock solution (dissolved in DMSO and 20% pluronic) for 30 min. Brain slices were then placed in the recording chamber and fixed with a U-shaped platinum wire with nylon strings. Brain slices were continuously perfused at a rate of 2 ml/min with ACSF that was gassed with carbogen (95 O_2_/5% CO_2_). Bath perfusion with ACSF was accomplished using a peristaltic pump (Vario, Ismatec, Germany). Drugs were applied via the perfusion system. If not stated otherwise, ACSF contained 1 μM TTX to suppress neuronal activity. Changes in intracellular calcium levels in olfactory bulb astrocytes were recorded by confocal microscopy (C1 Eclipse, Nikon, Düsseldorf, Germany). An excitation laser wavelength of 488 nm and a frame rate of 0.66 fps were used, and the fluorescence was collected through a 500–530 nm bandpass filter.

### Data Analysis and Statistic

The data were evaluated with Nikon EZ-C1 Viewer (Nikon) and statistical tests have been applied with Origin Pro 9.1 (OriginLab Corporation, Northampton, USA). To analyze changes of the calcium level in astrocytes, all cell somata were manually marked as regions of interests (ROIs), each ROI outlining the silhouette of the soma. Cells located in the GL and EPL that showed a calcium response to bath-applied ADP and to low extracellular K^+^ were identified as astrocytes (Hartel et al., 2007; Doengi et al., 2008; Fischer et al., 2020). The astrocytic calcium elevations have been observed in both GL and EPL without a significant difference in amplitude or duration, therefore the results in the present study are pooled data of both layers. To evaluate changes of calcium levels over time, Fluo-8 fluorescence intensity (F) was recorded during the course of the experiment and normalized to the basal fluorescence intensity in absence of pharmacological stimuli. Changes in calcium are given by ΔF as the percentage changes in fluorescence with respect to the basal fluorescence which was set to 100%. Since bleaching of Fluo-8 or slight movement of the tissue can lead to shifts in the baseline, the baseline was adjusted for each calcium response to the average of the last 10 data points before each calcium response. The starting time point of the response was defined as the first time point with a fluorescence value clearly higher than the baseline, which could easily be identified due to the rapid onset of the response. The peak of the calcium response was taken as the maximum value during the response. Calcium responses were assessed as amplitude (baseline to peak) and area (integral of the curve from the starting time point of the response until baseline was reached again). Duration of the response was assessed as the time span from the starting time point until the time point when the curve returned to baseline. All values are given as mean values ± standard error of the mean with “*n*” representing the sample size, given as “number of cells/number of brain slices/number of animals.” Some of the data populations were not normally distributed (Kolmolgorov-Smirnov's test), hence proof of statistical significance for paired data was done by non-parametric Wilcoxon signed-rank test at an error probability *p*: ^*^p < 0.05; ^**^p < 0.01; ^***^p < 0.001. In case of independent data the Mann-Whitney-U test was applied. Multiple comparisons were done using Kruskal-Wallis ANOVA followed by Dunn's *post-hoc* test (all tests done using Origin Pro 9.8, OriginLab Corp., Northampton, MA). To test the effect of a drug or combination of drugs (e.g., a receptor antagonists) on norepinephrine-evoked calcium signals in a test experiment, a first application of norepinephrine in the absence of the drug was performed, followed by a second application in the presence of the drug. Drugs were pre-incubated at least for 5 min. The effect of the drug was then expressed as percentage inhibition of the norepinephrine-evoked calcium response compared to the first application. However, in a rundown experiment, the second application of norepinephrine in the absence of a drug *per se* resulted in a significantly smaller calcium response (rundown) compared to the first application. The effect of the rundown was given as percentage reduction compared to the first application. A drug was only considered effective if the inhibition in the presence of the drug (second application, test experiment) was significantly larger than the reduction in the absence of the drug (second application, rundown experiment) which served as control. Therefore, means of the second application of test experiments were tested against the mean of the second application in the rundown experiment. In the figures, data populations are represented as violin plots. The violin plots also include symbols representing single data values, the median and 25–75% percentiles as horizontal lines as well as the mean values. Mean values of different data populations within a given graph are connected by a dotted line to improve visualization.

## Results

### Close Proximity of Noradrenergic Fibers and Astrocyte Processes

**Figure 1:**
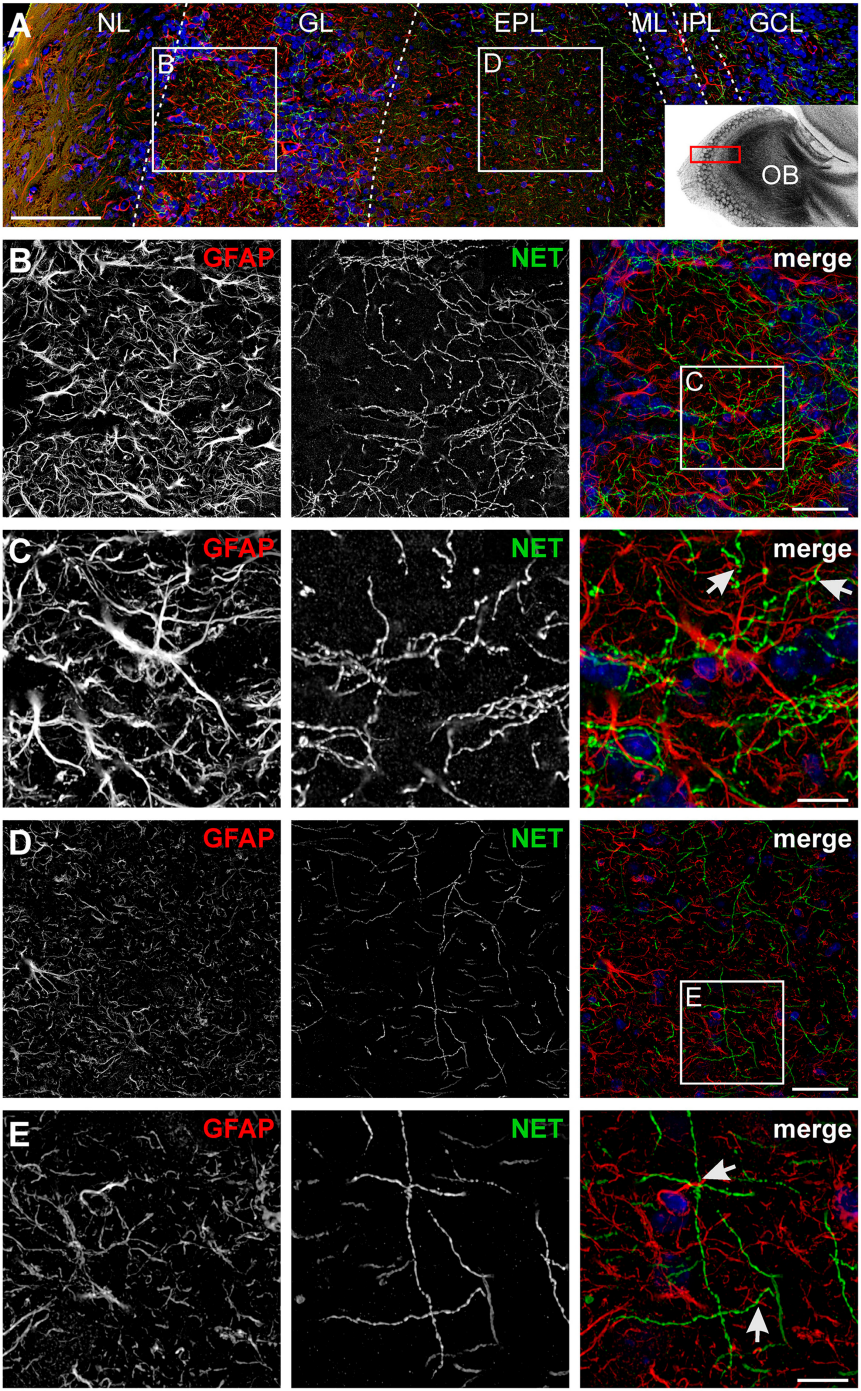
Visualization of noradrenergic fibers and astrocytes in the olfactory bulb. **(A)** Laminar organization of the olfactory bulb. Astrocytes were labeled by GFAP immunostaining (red), noradrenergic fibers were visualized by staining of the norepinephrine transporter (NET; green). Nuclei were stained with Dapi (blue). NL, nerve layer; GL, glomerular layer; EPL, external plexiform layer; ML, mitral cell layer; IPL, internal plexiform layer; GCL, granule cell layer. Scale bar: 100 μm. The inset depicts the location of the section in the olfactory bulb. **(B)** Astrocytes and noradrenergic fibers in the GL. Scale bar: 20 μm. **(C)** Magnified view of the GL. Astrocytes and noradrenergic fibers strongly intermingle, bringing astrocytic processes, and noradrenergic varicosities closely together (arrows). Scale bar: 5 μm. See also Supplementary Figure 1. **(D)** Astrocytes and noradrenergic fibers in the EPL. Scale bar: 20 μm. **(E)** Magnified view of the EPL. Arrows point to astrocyte processes adjacent to varicosities. Scale bar: 5 μm. All images represent projections of image stacks spanning 8 μm in depth.

We first studied the structural relationship between noradrenergic fibers originating in the LC and olfactory bulb astrocytes. Noradrenergic fibers were visualized using an antibody against norepinephrine transporters (NET) while astrocytes were highlighted using anti-GFAP staining (Figure 1). Both noradrenergic fibers and astrocytes were present in all layers of the olfactory bulb except the most superficial layer, the nerve layer. Sensory information is propagated by axons of olfactory sensory neurons that project into spherical neuropilar structures called glomeruli where the axons synapse onto projection neurons, mitral, and tufted cells. Glomeruli were penetrated by a dense meshwork of noradrenergic fibers that intermingle with astrocyte processes. Frequently, astrocyte processes were in close proximity of varicosities, the release sites of centrifugal fibers (Figures 1B,C; Supplementary Figure 1). In the EPL, the density of noradrenergic fibers was lower, however, astrocyte processes adjacent to varicosities were also found in the EPL (Figures 1D,E; Supplementary Figure 1). These results suggest that astrocytes might be able to detect norepinephrine released from centrifugal fibers of the LC.

### Norepinephrine Induces Calcium Transients in Olfactory Bulb Astrocytes

**Figure 2:**
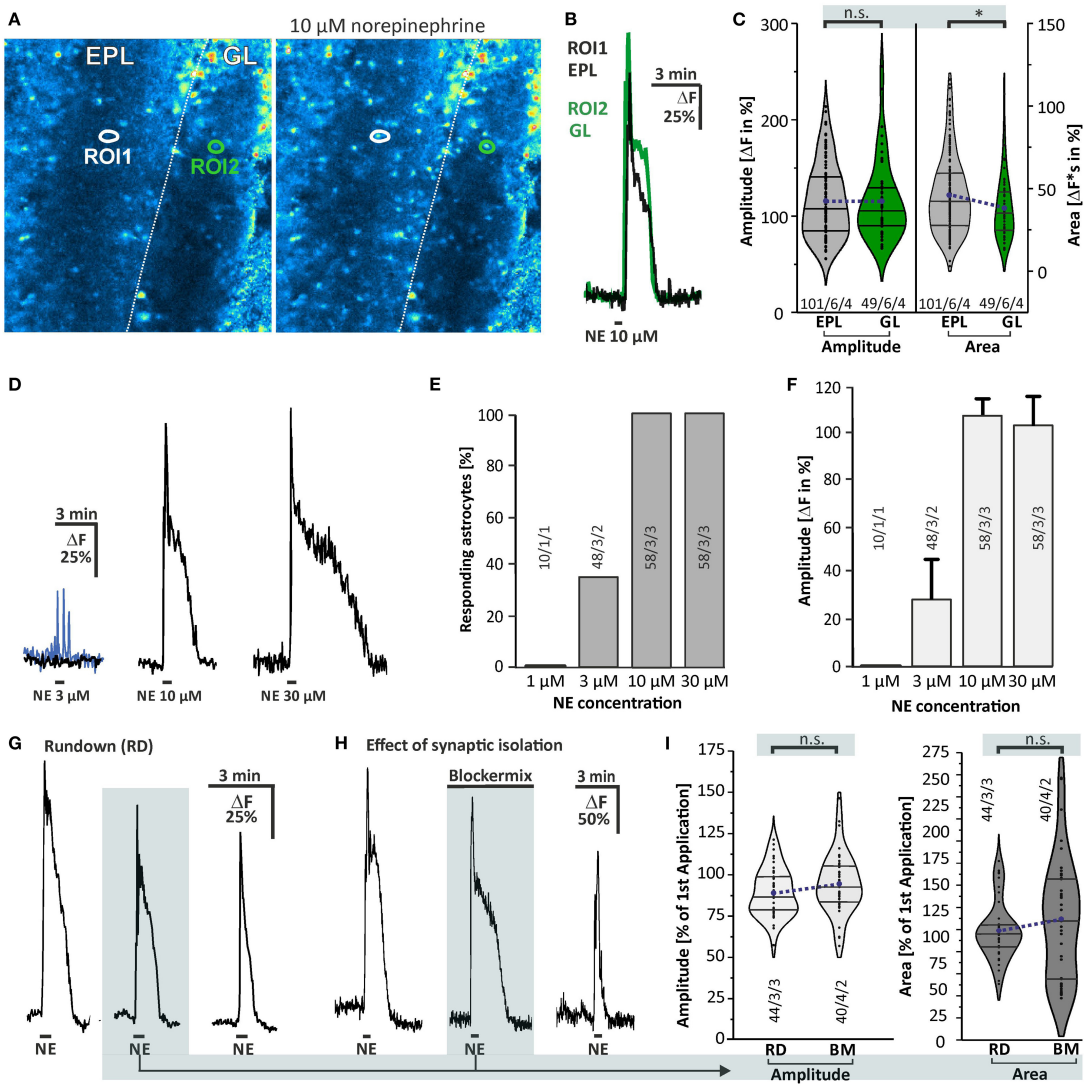
Norepinephrine-induced calcium transients in olfactory bulb astrocytes. **(A)** Fluo-8 staining of acute olfactory bulb slices with example ROIs shown in idle state (left) and in the presence of 10 μM norepinephrine (right). **(B)** Example traces of cells located in the external plexiform layer (EPL; ROI1, black trace) and in the glomerular layer (GL; ROI2, green trace). **(C)** Violin plots of amplitude (left) and area (right) of norepinephrine-induced calcium responses in EPL (gray) and GL (green) (see section Materials and Methods for details). Sample size as specified: cells/slices/animals. n.s., not significant. *p < 0.05. **(D)** Dose-response relationship of calcium transients evoked by norepinephrine (NE). At 3 μM NE, most astrocytes did not respond with Ca^2+^ transients (black trace), while some astrocytes generated a small Ca^2+^ rise (blue trace). **(E)** 1 μM NE failed to trigger calcium transients in astrocytes, while 3 μM evoked a Ca^2+^ response in about one-third of the astrocytes. Ten micromolars of NE was sufficient to induce calcium transients in all astrocytes investigated, with **(F)** maximal amplitude. **(G)** Example of multiple applications of NE (10 μM) with a 10-min interval displaying a significant signal rundown. **(H)** Calcium transients were not affected in presence of tertodotoxin (TTX), GABAergic, glutamatergic, and purinergic antagonists (Blockermix contains: NBQX 10 μM, D-APV 50 μM, gabazine 10 μM, CGP55845 10 μM, MPEP 2 μM, MRS2179 50 μM, ZM241385 0.5 μM, TTX 1 μM). For statistical analyses, the second application in blockermix was compared to the second application in the rundown experiment [depicted in **(G)**]. **(I)** Ca^2+^ responses by the second application of NE were normalized to responses of the first application, set to 100%. Amplitudes (left) and area (right) of calcium responses compared between the rundown experiment, i.e., in the absence of the blockermix (RD), and in presence of the blockermix (BM). Both amplitude and area were not significantly different (n.s.).

The present study focuses on the effect of norepinephrine on astrocytes in the GL and EPL of the olfactory bulb, the layers with the highest synaptic activity in the olfactory bulb (Rotermund et al., 2019). We used application of ADP to test the viability of the cells and to identify astrocytes in the olfactory bulb. Olfactory bulb astrocytes express P2Y1-receptors and respond to ADP with calcium transients, whereas earlier studies could show that Fluo-8-loaded olfactory bulb neurons do not respond to bath application of ADP (Doengi et al., 2008, 2009; Fischer et al., 2020). The aim of our study was to find out whether norepinephrine induces calcium transients in olfactory bulb astrocytes. We recorded calcium responses in astrocytes of the GL and EPL (Figures 2A,B). Application of 10 μM norepinephrine led to a cytosolic calcium rise with an amplitude of 115.0 ± 4.3% ΔF and an area of 47.5 ± 2.2 ΔF^*^s (*n* = 101/6/4) in the EPL and an amplitude of 116.1 ± 5.9% and area of 40.1 ± 2.8 ΔF^*^s (*n* = 49/6/4) in the GL, showing no significant difference in response amplitude and a small yet significant difference in the area (*p* = 0.025) between the layers (Figure 2C). Since the differences were neglectable we pooled data of the two layers for all following experiments. To appraise the optimal dose of norepinephrine we applied various concentrations. The results show that the minimal concentration to which all astrocytes responded was 10 μM, while some astrocytes started to respond to 3 μM (Figures 2D–F). The response consisted of a rapid increase that peaked after a few seconds, followed by a rapid decrease that turned into a plateau-like phase that stayed elevated at least as long as the application of norepinephrine continued (Figure 2B). Multiple applications of 10 μM norepinephrine in the same preparation led to a signal rundown, which resulted in a significant reduction by 10.8 ± 2.2% (*n* = 44/3/2; *p* < 0.001) in the second and 26.2 ± 2.6% (*n* = 44/3/2; *p* < 0.001) in the third application (Figure 2G) as compared to the first application (control). We refer to this as “rundown experiment.” We then compared responses evoked by norepinephrine application in the presence of a mix of GABAergic, glutamatergic, and purinergic antagonists (blockermix; GABAA: Gabazine 10 μM; GABAB: CGP55845 10 μM; AMPA-receptors: NBQX 10 μM; NMDA-receptors: D-APV 50 μM; mGluR5: MPEP 2 μM; P2Y1: MRS2179 50 μM; A2A: ZM241385 0.5 μM; sodium channels: TTX 1 μM) with the corresponding application in the absence of receptor antagonists (second application, rundown experiment) to evaluate the contribution of these receptors to the norepinephrine-evoked response and to isolate the response from indirect neuronal impact (Figures 2H,I). In the presence of the blockermix, no significant differences between the values of the second application in the control experiments and in experiments using the blockermix were found, indicating that the norepinephrine-evoked calcium signals were not modulated by spontaneous neuronal activity or synaptic transmission as they were not affected by application of the blockermix (Figure 2I). We nevertheless performed all subsequent experiments in the presence of this blockermix to suppress any indirect neuronal effect. Hence, the second application of the experiments in the presence of the blockermix served as control for the following experiments.

### Norepinephrine-Induced Calcium Transients Depend on Both α1 and α2, but Not on β-receptors

**Figure 3:**
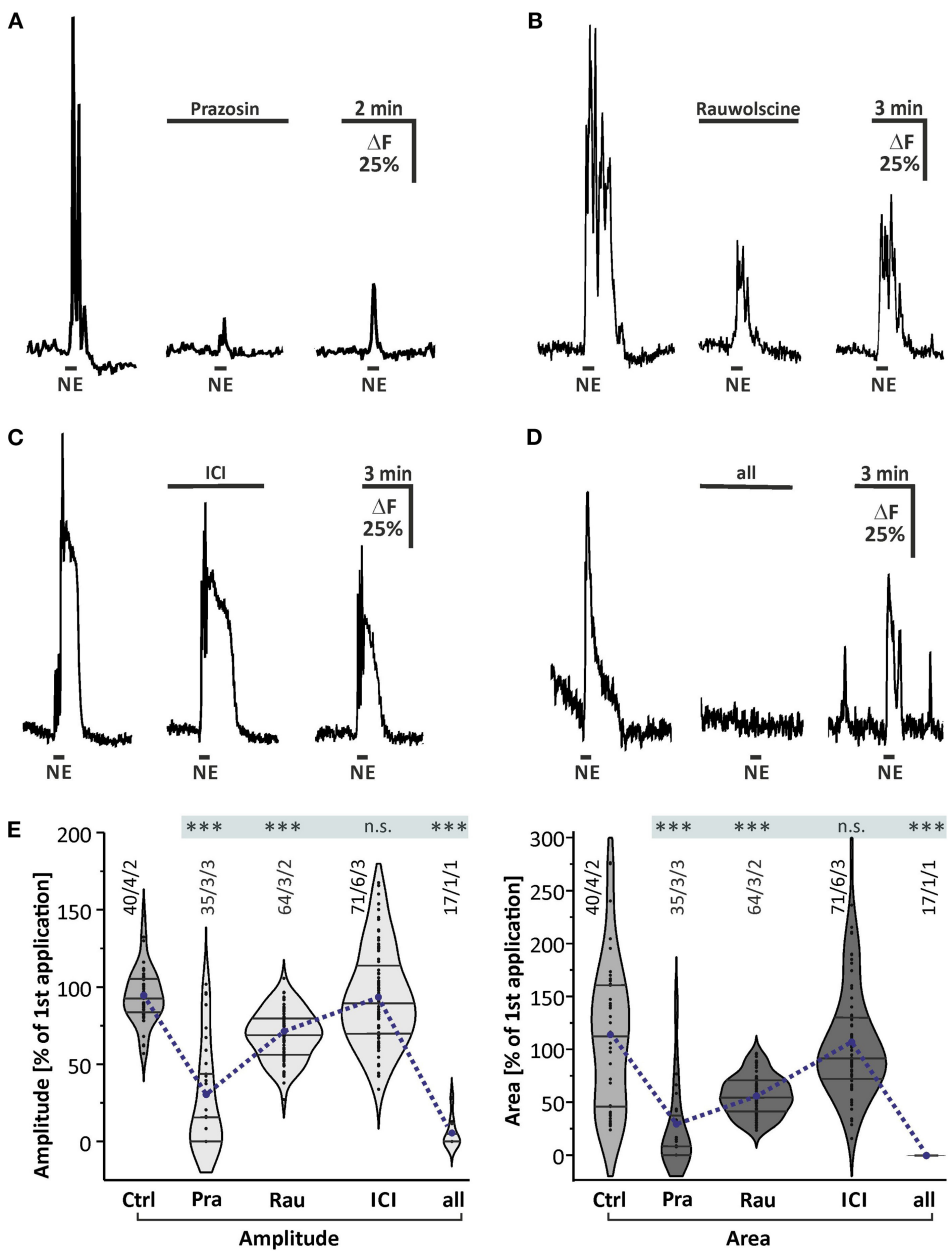
Effect of noradrenergic receptor antagonists. **(A)** All experiments were performed in the presence of the blockermix described in Figure 2 to isolate the direct response in astrocytes. Norepinephrine-induced calcium responses were strongly reduced in the presence of α1-antagonist prazosin (10 μM). **(B)** Calcium transients were affected in the presence of the α2-antagonist rauwolscine (0.5 μM). **(C)** Calcium transients were not significantly affected by β-receptor antagonist ICI-118,551 (15 μM). **(D)** Calcium transients were entirely abolished in the presence of a combination of all antagonists mentioned above. **(E)** Violin plots of amplitudes (light gray) and area (dark gray) of calcium responses under control conditions (i.e. second application of the blockermix experiment depicted in Figure 2H) and in the presence of prazosin (Pra), rauwolscine (Rau), ICI, and a combination of all. n.s. = not significant; ***P < 0.001.

Taking into account that norepinephrine-evoked calcium transients in astrocytes were not affected by blockers of neuronal voltage-gated sodium channels and neurotransmitter receptors, we assumed that astrocytes expressed noradrenergic receptors themselves. Thus, we applied norepinephrine in the presence of antagonists for the major subtypes of noradrenergic receptors to test whether the norepinephrine-induced calcium transients are affected (Figure 3). In the presence of the α1-receptor antagonist prazosin, norepinephrine-induced calcium transients were reduced by 65.3 ± 6.8% (*n* = 35/3/3; *p* < 0.001) in amplitude and 70.9 ± 7.1% (*n* = 35/3/3; *p* < 0.001) in area (Figures 3A,E). Under the influence of the α2-antagonist rauwolscine, the norepinephrine-induced calcium transients were diminished by 27.5 ± 2.6% (*n* = 64/3/2; *p* < 0.001) in amplitude and 43.1 ± 6.2% (*n* = 64/3/2; *p* < 0.001) in area (Figures 3B,E). To test the possible involvement of β-adrenergic receptors, we used the β-selective antagonist ICI-118,551. In the presence of ICI-118,551, norepinephrine-induced calcium transients were not significantly reduced in amplitude and in area when compared to the second application of the control experiment (*n* = 71/6/3; *p* > 0.05) (Figures 3C,E). In the presence of all antagonists mentioned above, the calcium transients were nearly completely abolished with a reduction of 94.6 ± 2.3% (*n* = 17/1/1; *p* < 0.001) in amplitude and 96.8 ± 1.1% (*n* = 17/1/1; *p* < 0.001) in area (Figures 3D,E).

### Norepinephrine-Induced Calcium Transients Depend on Intracellular Calcium Stores

**Figure 4:**
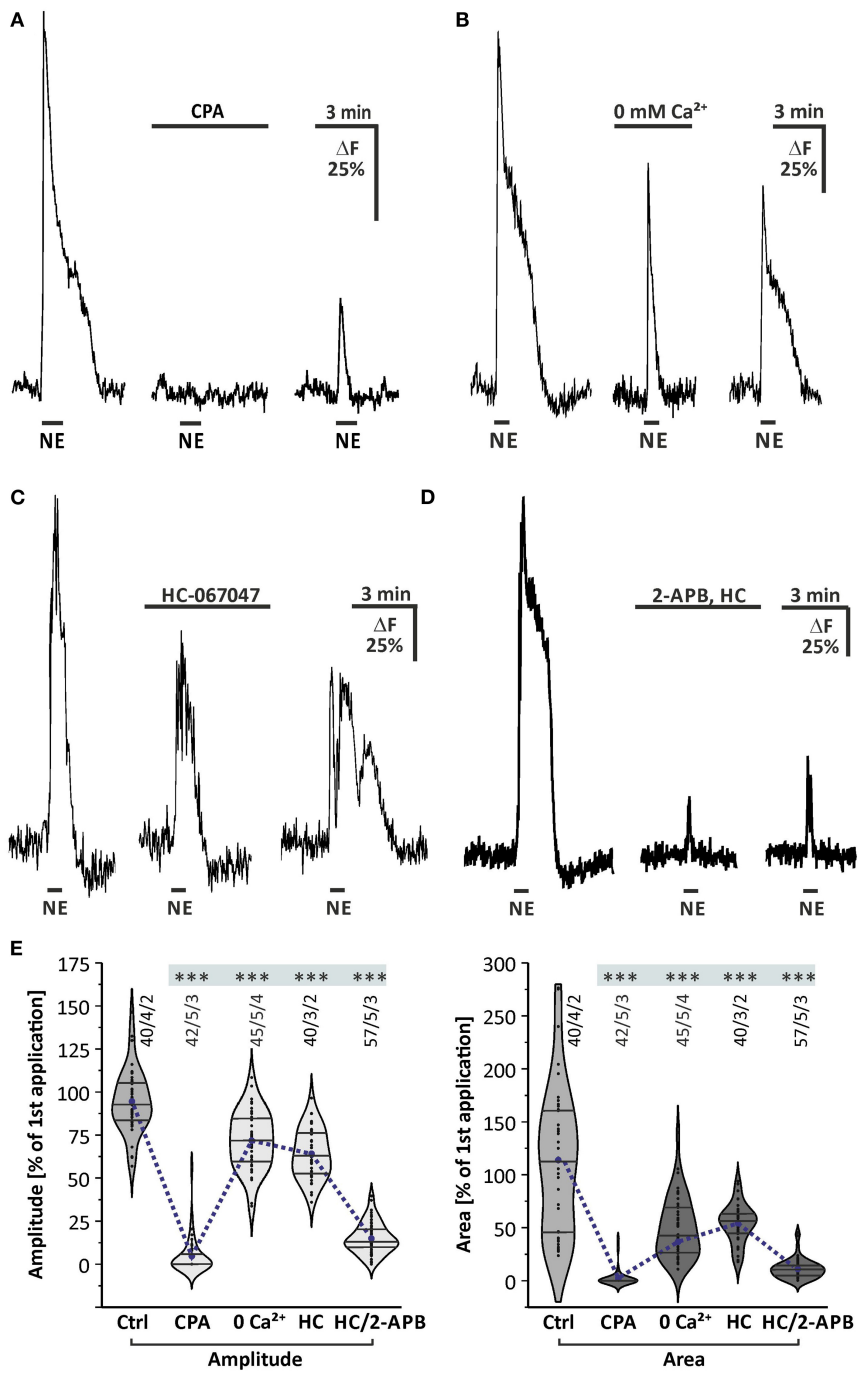
Internal calcium release and phospholipase C mediate norepinephrine-induced calcium transients in olfactory bulb astrocytes. **(A)** Norepinephrine-induced calcium transients are entirely suppressed in the presence of SERCA inhibitor cyclopiazonic acid (CPA, 20 μM). **(B)** Calcium transients are significantly reduced in the absence of extracellular calcium (0 mM Ca^2+^). **(C)** Norepinephrine-induced calcium transients are partly suppressed in the presence of TRPV4 inhibitor HC-067047 (1 μM). **(D)** Calcium response is entirely abolished in the presence of IP3-receptor antagonist 2-APB (100 μM) and HC-067047 (HC). **(E)** Violin plots of amplitudes (light gray) and area (dark gray) of calcium responses under control conditions (i.e., second application of the blockermix experiment depicted in Figure 2H) and in the presence of CPA, Ca^2+^-free solution, HC-067047 (HC) alone and in combination with 2-APB. ***P < 0.001.

We next aimed to investigate whether the cytosolic calcium rise depends on intracellular calcium stores. To elucidate this, we applied norepinephrine before and after calcium stores were depleted by incubation with 20 μM cyclopiazonic acid (CPA), an inhibitor of the sarco-/endo-plasmic reticulum calcium-ATPase (SERCA). Cyclopiazonic acid entirely suppressed norepinephrine-evoked calcium responses (Figure 4A). The mean amplitude of norepinephrine-induced calcium transient was reduced by 95.5 ± 1.6% (*n* = 42/5/3; *p* < 0.001) in amplitude and by 97.2 ± 2.7% (*n* = 42/5/3; *p* < 0.001) in area (Figures 4A,E), indicating that release of calcium from the endoplasmic reticulum (ER) is required for the norepinephrine-induced cytosolic calcium rise. We also tested the contribution of extracellular calcium to norepinephrine-evoked calcium responses. Withdrawal of extracellular calcium led to a reduction by 29.2 ± 3.2% (45/5/4; *p* < 0.001) in amplitude of norepinephrine-evoked calcium responses, while the area of the response was reduced by a much larger fraction of 66.2 ± 3.9% (45/5/4; *p* < 0.001) (Figures 4B,E). This indicates that norepinephrine-induced calcium transients not exclusively depend on intracellular calcium, but also needs calcium influx from the extracellular space. In particular, the late plateau-like phase of the response was entirely suppressed in the absence of extracellular calcium, suggesting that the plateau was mediated by calcium influx.

To test whether the norepinephrine-induced calcium rise depends on the PLC/IP3 signaling pathway, we applied norepinephrine after incubation with the IP3 receptor antagonist 2-APB. Since concentrations above 50 μM of 2-APB activate transient receptor potential channels TRPV and lead to calcium oscillations in olfactory bulb astrocytes (Doengi et al., 2009), the selective TRPV4 antagonist HC-067047 (1 μM) was co-applied with 2-APB to suppress TRPV4-mediated calcium oscillations and to allow for a higher concentration of 2-APB. However, HC-067047 alone partly reduced the amplitude and area of norepinephrine-evoked calcium responses (Figures 4C,E). Additional application of 100 μM 2-APB almost entirely suppressed norepinephrine-induced calcium responses, on average by 83.9 ± 1.6% (*n* = 57/5/3; *p* < 0.001) in amplitude and 90.3 ± 2.5% (*n* = 57/5/3; *p* < 0.001) in area, indicating the involvement of IP3-receptors (Figure 4E). It must be noted, however, that 2-APB also blocks calcium channels such as Orai and some TRP channels and hence part of the effect of 2-APB is on calcium influx from the extracellular space (see below).

### Store-Operated Calcium Entry in Olfactory Bulb Astrocytes

**Figure 5:**
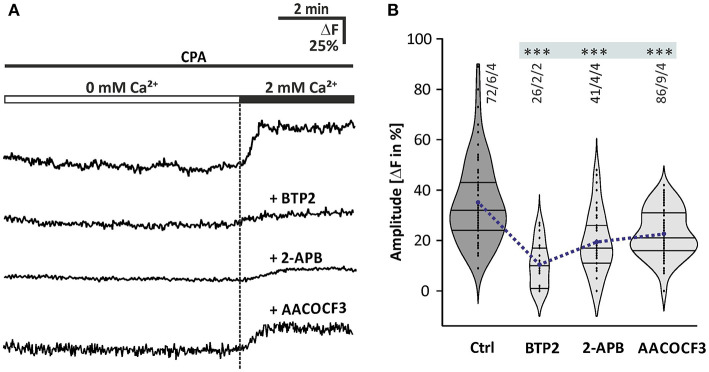
Store-operated calcium entry in olfactory bulb astrocytes. **(A)** Original recordings of the cytosolic calcium concentration in somata of astrocytes of four different experiments. Acute brain slices were exposed to 20 μM CPA for 10 min in calcium-free solution (0 mM Ca^2+^) before 2 mM calcium was re-added, and the resulting cytosolic calcium rise was indicative of SOCE. Traces show astrocytes that had been treated with CPA alone as a control, and with CPA in combination with the SOCE channel inhibitors 2-APB (100 μM) and BTP2 (25 μM) as well as the iPLA2 inhibitor AACOCF3 (25 μM). **(B)** Violin plots of amplitudes of calcium responses after calcium re-addition under control conditions (only CPA) and in the presence of 2-APB, BTP2, and AACOCF3. ***P < 0.001.

It is not yet known which mechanism is responsible for calcium influx from the extracellular space into olfactory bulb astrocytes. Since calcium is partly transported out of the cell by plasma membrane calcium transporters, glial cells accomplish calcium store refilling by employing plasma membrane calcium channels under the control of the ER calcium content, a mechanism known as SOCE (Parekh and Putney, 2005). It has been shown that SOCE plays an important role in calcium homeostasis and signaling of cerebellar glial cells (Singaravelu et al., 2006). To determine whether SOCE is relevant in olfactory bulb astrocytes, we studied SOCE as induced by CPA in olfactory bulb astrocytes regarding its sensitivity to different drugs (Figure 5). The CPA-induced depletion of calcium stores resulted in calcium transients during re-addition of calcium with a mean amplitude of 35.1 ± 1.9% ΔF (*n* = 72/6/4; *p* < 0.001). The calcium transients were significantly reduced by 45 ± 1.9% (*n* = 41/4/4; *p* < 0.001) in the presence of 2-APB (100 μM), an inhibitor of SOCE channels (Ma et al., 2000; Bakowski et al., 2001; Singaravelu et al., 2006). 2-Aminoethyl diphenylborinate was applied 10 min before the re-addition of calcium in the presence of CPA (Figures 5A,B). However, since 2-APB is also an inhibitor of PLC (see Figure 4), we performed experiments using BTP2, a structurally distinct blocker of SOCE channels also known as calcium release-activated channels (CRAC), but without effect on PLC (Ishikawa et al., 2003; Zitt et al., 2004). BTP2 (25 μM), pre-incubated for 10 min, significantly reduced SOCE by 62.9 ± 3.1% (*n* = 26/2/2; *p* < 0.001) (Figures 5A,B). To test whether iPLA2 plays a role in SOCE in olfactory bulb astrocytes, as it has been shown for Bergmann glia and cerebellar granule cells (Singaravelu et al., 2006, 2008), a specific inhibitor of iPLA2, AACOCF3 (25 μM) was applied. It is well-established to inhibit iPLA2 at a concentration of 15 μM and higher (Ackermann et al., 1995). The calcium transients during calcium re-addition in the presence of CPA were significantly reduced by 35.4 ± 1.0% ΔF (*n* = 86/9/4; *p* < 0.001) by AACOCF3 (Figures 5A,B). Our results show that depletion of calcium stores evokes SOCE, involving ICRAC channels and iPLA2.

### Store-Operated Calcium Entry Contributes to Norepinephrine-Induced Calcium Transients

**Figure 6:**
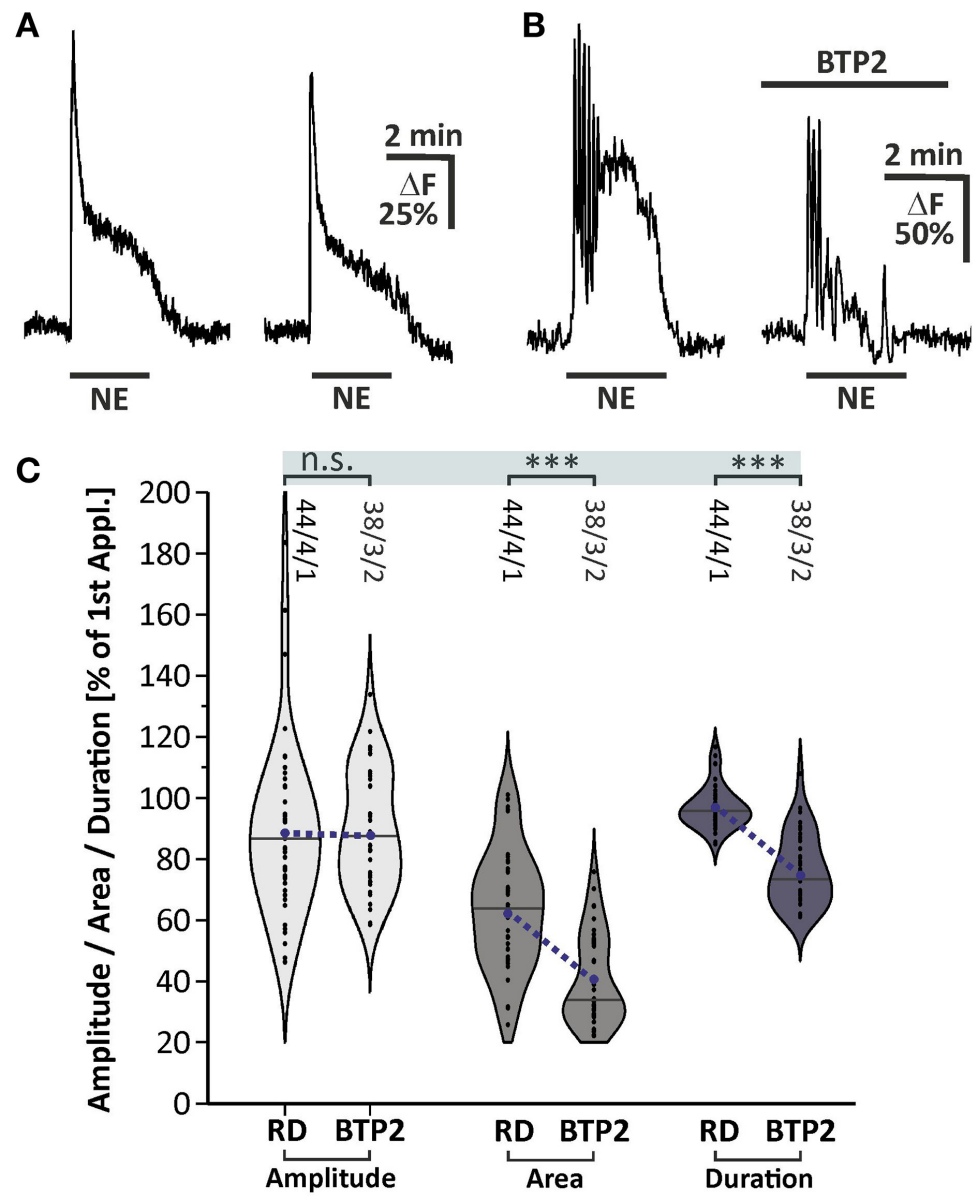
Store-operated calcium entry contributes to norepinephrine-induced calcium transients in olfactory bulb astrocytes. **(A)** Example of two consecutive applications of norepinephrine (NE, 10 μM) for 150 s with a 10-min interval. **(B)** Calcium transients were affected in area and duration by BTP2. **(C)** Violin plots of amplitudes (light gray), area (mid gray) and duration (dark gray) of calcium responses under control conditions [i.e. second application of the rundown experiment depicted in **(A)**] and in the presence of BTP2. n.s. = not significant, ***P < 0.001.

We investigated the involvement of SOCE in norepinephrine-induced calcium transients. The late plateau-like phase of the norepinephrine-evoked calcium response was abolished in calcium-free solution, indicating calcium influx (Figure 4B). To elucidate whether SOCE is the crucial mechanism responsible for this late phase calcium influx contributing to the norepinephrine-induced calcium response, we applied norepinephrine in the presence of the SOCE inhibitor BTP2 (25 μM). For this experiment, the duration of application was extended to 150 s to elongate the late phase of the calcium response. The data obtained were compared with a rundown experiment adapted to the modified application time of norepinephrine (Figure 6A). The second application of norepinephrine in the rundown experiment induced a calcium response with an amplitude of 89.0 ± 4.0% (*n* = 44/4/1) and area of 63.3 ± 2.9% (*n* = 44/4/1) compared to the first application (Figure 6A). Addition of BTP2 had no significant effect on the amplitude of the calcium response (*n* = 38/3/2), indicating that the initial Ca^2+^ peak is not mediated by BTP2-sensitive Ca^2+^ influx (Figures 6B,C). In contrast, the area of the second response was significantly reduced by BTP2 (*n* = 38/3/2; *p* < 0.001), indicating a prominent role of SOCE in the late phase of norepinephrine-induced calcium transients in olfactory bulb astrocytes. In addition, the Ca^2+^ trace returned to baseline even in the presence of norepinephrine (Figure 6B) and thus the duration of the calcium response was significantly shortened by BTP2 (*n* = 38/3/2; *p* < 0.001) (Figure 6C). In summary, norepinephrine evokes a biphasic calcium response in olfactory bulb astrocytes, consisting of an initial and transient calcium peak reflecting internal calcium release, followed by a sustained plateau phase established by calcium influx via SOCE channels.

## Discussion

### Norepinephrine Induces Calcium Transients in Olfactory Bulb Astrocytes

In the present study, we showed that norepinephrine induced a rise in cytosolic calcium concentration in mouse olfactory bulb astrocytes *in situ*. The study was performed on brain slices obtained from animals at an age of 1–3 weeks and we cannot exclude changes in adrenergic calcium signaling in olfactory bulb astrocytes during adolescent development and aging. Ten micromolars of norepinephrine were needed to reliably trigger calcium responses, which appears to be rather high. However, norepinephrine is rapidly taken up by NET that are abundant in the olfactory bulb according to our immunhistological stainings. In addition, extracellular norepinephrine is degraded by catechol-O-methyltransferase (COMT), an enzyme with particular high expression and efficacy in the olfactory bulb (Eisenhofer et al., 2004; Cockerham et al., 2016). Hence, the effective concentration of norepinephrine at the receptor most likely was drastically less than adjusted in the perfusion solution. We demonstrated that the norepinephrine-induced calcium rise was entirely blocked in the presence of the SERCA inhibitor CPA, indicating that norepinephrine triggers calcium release from internal stores such as the ER. In the absence of extracellular calcium, on the contrary, the norepinephrine-induced calcium transients were only slightly reduced in amplitude, but to a larger extend in area and duration, indicating an involvement of extracellular calcium at least in the late plateau-like phase of the calcium response. The most common mechanism to liberate calcium from the ER is the phospholipase C (PLC)/IP3 signaling pathway, which has previously been shown for olfactory bulb astrocytes to be activated by P2Y1, A2A, mGluR5, and dopamine receptors and has been confirmed in the present study (Doengi et al., 2008; Otsu et al., 2015; Fischer et al., 2020). Calcium withdrawal suppressed only the late plateau phase of the calcium response with little effect on the initial calcium peak, whereas suppression of internal calcium release by CPA entirely blocked norepinephrine-mediated calcium responses. This suggests that calcium influx is downstream to calcium release, in line with SOCE which is only activated after calcium store depletion (Lopez et al., 2020). Indeed, the late calcium influx could be blocked by the SOCE channel blockers BTP2 and 2-APB (Somasundaram et al., 2014). Involvement of SOCE in norepinephrine-induced calcium transients has also been shown for hippocampal and cortical astrocytes (Gaidin et al., 2020; Okubo et al., 2020). Furthermore, we demonstrate that SOCE is dependent of iPLA2 by suppressing iPLA2 activity with AACOCF3, as shown in cerebellar astrocytes and granule cells as well as olfactory bulb ensheathing cells (Singaravelu et al., 2006, 2008; Thyssen et al., 2013).

### α1 and α2-Adrenergic Receptors Mediate Calcium Signaling in Olfactory Bulb Astrocytes

The different subtypes of norepinephrine receptors are known to be linked to different signaling cascades. For α1-receptors the Gq-coupling was shown early on (Schwinn, 1993). α2-receptors, on the contrary, are mainly known to be negatively coupled to adenylate cyclase activity via Gi-proteins, but recent studies revealed a α2-receptor-mediated and Gq-coupled calcium response in hippocampal astrocytes (Gaidin et al., 2020). The norepinephrine-induced cytosolic calcium rise was decreased by α1- and α2-receptor antagonists, but not by a β-antagonist, indicating that both α-receptor subtypes contribute to norepinephrine-evoked calcium transients. There is a large amount of studies that have investigated adrenoceptor expression and function in astrocytes. Early electron microscopic studies on cortex first demonstrated both α- and β-adrenergic receptors in astrocytic membranes (Aoki, 1992; Aoki et al., 1998) and, moreover, a close proximity of astrocytic processes with LC-noradrenergic varicosities and terminals (Cohen et al., 1997; Aoki et al., 1998), suggesting that astrocytes are well-positioned as a target for norepinephrine release from LC fibers. Our immunohistological results suggest that in the olfactory bulb astrocytic processes and noradrenergic varicosities are in close proximity. Astrocytes have also been shown to respond to norepinephrine *in situ* with calcium transients in hippocampal brain slices, mediated primarily by α1-adrenergic receptors (Duffy and MacVicar, 1995). More recently, α1-receptors have been highlighted as important participants in astrocytic calcium signaling in many different brain regions, such as cerebellum, ventrolateral medulla, and visual cortex (Ding et al., 2013; Paukert et al., 2014; Schnell et al., 2016). Furthermore, it has been demonstrated in awake behaving mice that α1-receptors of layer 2/3 neocortical astrocytes are very sensitive to norepinephrine and can induce gliotransmission of ATP and D-serine. Gliotransmission is followed by ATP receptor-mediated currents in adjacent pyramidal neurons which contributes to neocortical synaptic plasticity (Pankratov and Lalo, 2015). Another recently discovered example of norepinephrine-induced gliotransmission is mediated by α1-receptors (Gaidin et al., 2020). However, there are also results suggesting that β-receptors may also be involved in astrocytic calcium signaling in the cortex (Bekar et al., 2008).

### Astrocytes as Neuromodulatory Elements in the Olfactory Bulb

In olfactory bulb neurons, the distribution of noradrenergic receptors has been extensively studied (Jahr and Nicoll, 1982; Trombley and Shepherd, 1992; Jiang et al., 1996; Matsutani and Yamamoto, 2008). Studies of the last decades on norepinephrine focused on the modulation of dendro-dendritic inhibition at the mitral cell–granule cell synapse with steadily increasing details (Nai et al., 2009, 2010; Linster et al., 2011; Li et al., 2015; Manella et al., 2017). For olfactory bulb astrocytes, the current work provides the first evidence for adrenergic receptors. However, norepinephrine is not the only neuromodulator that evokes calcium signaling in olfactory bulb astrocytes, yet it is the first “top down” neuromodulator released from centrifugal fibers described so far to act on astrocytes in the olfactory bulb. Dopamine is a neuromodulator that is released by local interneurons in the olfactory bulb and induced calcium responses in astrocytes similar to those evoked by norepinephrine (Fischer et al., 2020). In addition, ATP is released from nerve terminals of olfactory sensory neurons in the GL and is degraded by ectoenzymes to adenosine, another neuromodulator inducing calcium signaling in olfactory bulb astrocytes (Doengi et al., 2008; Thyssen et al., 2010; Thyssen et al., 2010). Interestingly, many of the neuromodulator receptors in astrocytes such as α2 adrenergic receptors, D1- and D2-like dopamine receptors and A_2A_ adenosine receptors that typically mediate stimulation or inhibition of adenylate cyclase are linked to IP_3_ receptor-dependent release of calcium from internal stores in olfactory bulb astrocytes. Such calcium signaling may have a significant impact on neuronal performance in the olfactory bulb on different levels. Upon mechanical stimulation, e.g., rat olfactory bulb astrocytes release GABA and glutamate to evoke ionic currents in mitral and granule cells (Kozlov et al., 2006). Another gliotransmitter released by olfactory bulb astrocytes is ATP that is degraded to adenosine, a potent neuromodulator in the olfactory bulb (Roux et al., 2015; Schulz et al., 2017; Rotermund et al., 2018). Besides gliotransmission, astrocytes contribute to the blood brain barrier, mediate neurovascular coupling, and hence adjust local blood flow (Petzold et al., 2008; Doengi et al., 2009; Otsu et al., 2015; Beiersdorfer et al., 2020). Olfactory bulb astrocytes not only transmit information to neurons and blood vessels, but also to olfactory ensheathing cells, another type of glial cell that is coupled to astrocytes in a panglial network (Beiersdorfer et al., 2019). Considering the increasing evidence for functional contribution of astrocytes to neuromodulation and neurovascular coupling in the olfactory bulb, it is likely that noradrenergic fibers not only affect neuronal performance by directly stimulating neuronal adrenoceptors, but also employ astrocytes as active components of neural circuits.

## Data Availability Statement

The raw data supporting the conclusions of this article will be made available by the authors, without undue reservation.

## Ethics Statement

The animal study was reviewed and approved by Behörde für Gesundheit und Verbraucherschutz, Hamburg, Germany.

## Author Contributions

TF, NR, and CL designed the study. TF, JP, and LE performed the experiments and analyzed the data. TF and CL wrote the manuscript. All authors contributed to the article and approved the submitted version.

## Conflict of Interest

The authors declare that the research was conducted in the absence of any commercial or financial relationships that could be construed as a potential conflict of interest.
