# Supplementary material for: Norepinephrine-Induced Calcium Signaling and Store-Operated Calcium Entry in Olfactory Bulb Astrocytes
Source: Front Cell Neurosci. 2021 Mar 23;15:639754. doi: 10.3389/fncel.2021.639754 (PMC8021869; doi:10.3389/fncel.2021.639754)
Supplement: Supplementary file 1 [file Data_Sheet_1.PDF]

Supplementary Material

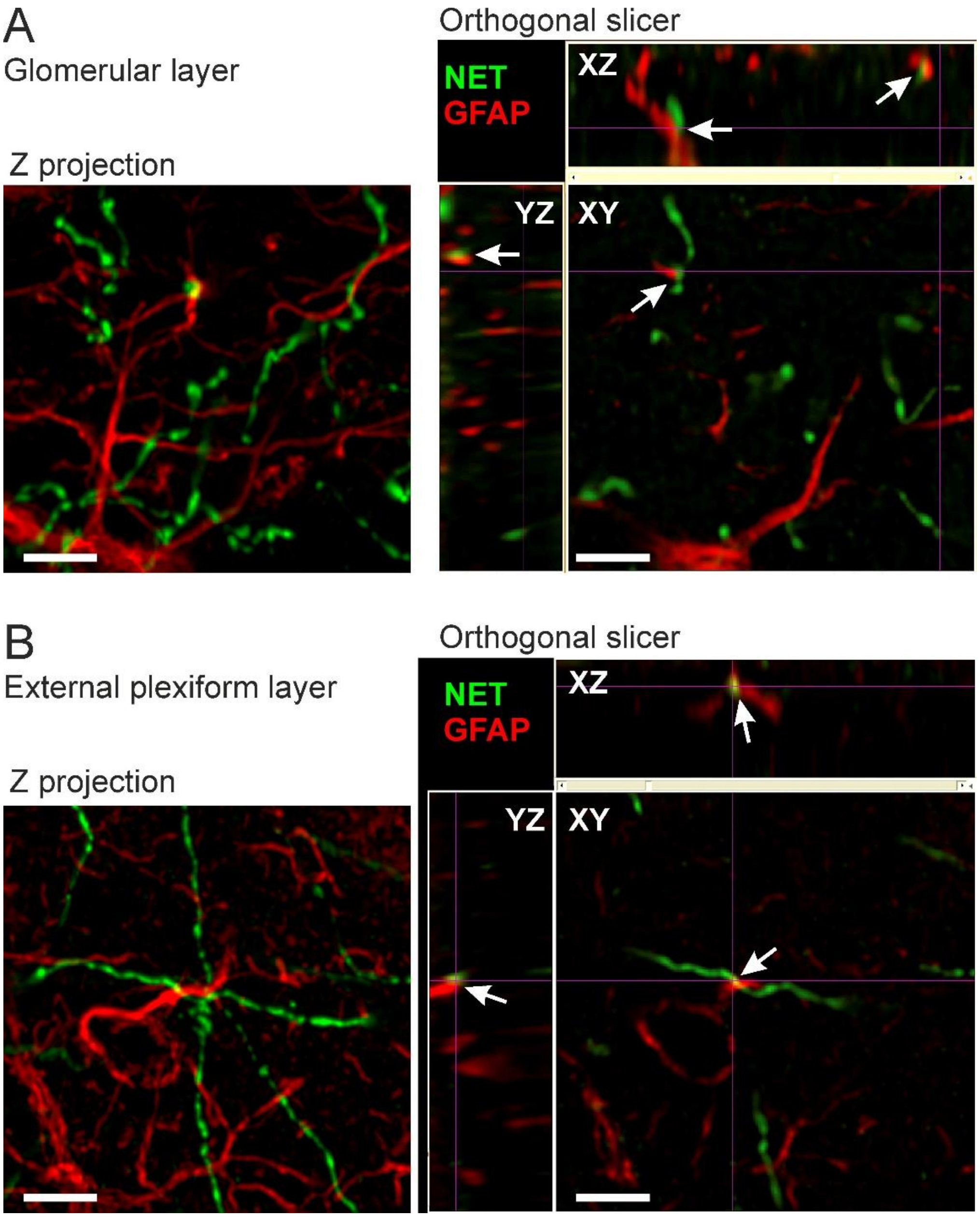

Supplementary Figure 1. Close proximity of noradrenergic fibers stained for norepinephrine transpoter NET (green) and astrocyte processes stained for GFAP (red) in the glomerular layer (A) and external plexiform layer (B). Z projections give an overview of the entire structures, while the orthogonal slicer provides spatial information in single plains in XY, XZ and YZ orientation. Arrows indicate NET-positive fibers adjacent to astrocyte processes. Scale bars: 5 µm.
